# Traumatic Brain Injury Guideline Implementation at a University Hospital in Saudi Arabia

**DOI:** 10.3390/life15030369

**Published:** 2025-02-26

**Authors:** Nawaf AlShahwan, Saleh Husam Aldeligan, Abdulaziz S. AlQahtani, Moath Shaher Sallam, Abdulaziz Alqusiyer, Ahmed A. Fallatah, Ahmed Alburakan, Hassan Mashbari, Abdullah Albdah, Thamer Nouh

**Affiliations:** 1 Trauma and Acute Care Surgery Unit, Department of Surgery, College of Medicine, King Saud University, P.O. Box 2925, Riyadh 11461, Saudi Arabia; 2 College of Medicine, King Saud University, P.O. Box 2925, Riyadh 11461, Saudi Arabia; 3 General Surgery Section, Department of Surgery, King Abdulaziz Hospital, Ministry of Health, P.O. Box 3665, Jeddah 22421, Saudi Arabia; 4 Trauma and Acute Care Surgery Fellow, Department of Surgery, College of Medicine, King Saud University, P.O. Box 2925, Riyadh 11461, Saudi Arabia; 5 Department of Surgery, Faculty of Medicine, Jazan University, P.O. Box 6809, Jazan 82817-28204, Saudi Arabia; 6 Trauma and Acute Care Surgery, Dr. Suliman Alhabib Medical Group, Sahafa Hospital, Riyadh 14212, Saudi Arabia

**Keywords:** traumatic brain injury (TBI), intracranial pressure (ICP) monitoring, practice management guidelines, brain trauma foundation (BTF), intensive care units (ICU)

## Abstract

Traumatic Brain Injuries (TBIs) pose a significant global health burden, with high mortality and long-term disability rates. This retrospective cohort study investigates the implementation and impact of the Brain Trauma Foundation (BTF) guidelines on TBI management in a Saudi Arabian tertiary care center. Data from the pre-implementation (2012) and post-implementation (2022) of BTF guidelines periods were compared. The patient demographics, injury severity, guideline adherence, and outcomes were analyzed. The results revealed a 33% increase in severe TBI cases from 2012 to 2022, possibly due to the development of a well-established trauma system designed to manage such cases. More importantly, implementing the BTF guidelines led to a marked improvement in patient outcomes. Compliance with guidelines, including the avoidance of hypoxia and hypotension, hyperosmolar therapy, and DVT prophylaxis significantly increased post-implementation. Mortality rates decreased from 45.4% to 14.2%, accompanied by a reduction in the intensive care unit (ICU) length of stay and an increase in the hospital length of stay. A comparison with international trauma centers highlights the importance of protocol-based management in improving the TBI outcomes and reducing the mortality—factors such as specialist-led care, institutional policies, and increased resources influenced guideline adherence and patient care. This study underscores the critical role of evidence-based guidelines in optimizing TBI management and emphasizes the need for their widespread implementation in clinical practice. In conclusion, this study demonstrates the substantial impact of BTF guidelines on enhancing TBI patient outcomes in a Saudi Arabian tertiary care center. The implementation of these guidelines resulted in a significant reduction in mortality and in an improved adherence to the best practices in TBI management, highlighting the importance of evidence-based protocols in optimizing patient care and outcomes.

## 1. Introduction

Traumatic Brain Injuries (TBIs) are a worldwide, widespread cause of death and long-term disability [1,2,3] as the mortality rate of patients with severe TBI is reported to be 30–50%, making severe TBI the leading cause of death below the age of 40 [3]. Annually, in the United States TBIs affect around 1.4 million people; around 3.5% of them die and 6% suffer from long-term disabilities [4,5]. Furthermore, during a population-based study conducted in San Diego in 1993, it was estimated that almost half of the deaths caused by trauma are due to CNS injuries [1]. In comparison, in Saudi Arabia, TBI incidence is 116 per 100,000 of the population, out of which the most common causes are road traffic accidents (RTA) (49.7%) [6]. Moreover, trauma is an important public health problem in Saudi Arabia. In its 2012 report, the Saudi Ministry of Health (MOH) reported that deaths due to injury, poisoning, and external causes were the second leading cause of death, accounting for 21.2% of all deaths in the country [7]. Despite that, the most recent annual report of the Saudi MOH reports a 35% decrease in all RTAs compared to the last five years. The development of a trauma system in our center decreased the severity of TBI cases, due to the efforts being made to save the patients’ lives, which were previously hindered due to the lack of expertise and facilities. Moreover, the Kingdom of Saudi Arabia is on its way to achieving the targeted (50%) decline in RTAs by 2030 [8]. Nonetheless, TBIs constitute an important part of this problem, necessitating improvements in prevention, acute care, and rehabilitation [9,10]. In 2007, the Brain Trauma Foundation (BTF) published the third edition of “Guidelines for the Management of Severe Traumatic Brain Injury” [9]. These guidelines were updated in 2016, with each item in the guidelines being changed or edited [10]. These guidelines address the key issues reducing the secondary injuries resulting from reduced perfusion to the brain. The high mortality rates have been reported to significantly drop over the years, with the implementation of the evidence-based protocols for the management of severe TBIs [11,12,13,14], and trauma centers in the United States are showing a dramatic increase in the adherence to these guidelines [15]. Moreover, a 2010 report by Arabi et al. showed for the first time that the implementation of a TBI clinical practice guideline, based on the BTF guidelines, led to a significant reduction in TBI mortality in their 800-bed trauma center in Riyadh, Saudi Arabia. Despite that, there is little data regarding BTF guideline implementation in TBI patients in intensive care units (ICU) in Saudi Arabia; therefore, in this paper, our aim is to examine the degree of the adaptation to the BTF’s “Guidelines for the Management of Severe Traumatic Brain Injury” in our university hospital’s surgical ICU. Furthermore, we aim to compare and outline the results of the implementation of the guidelines in 2012 and 2022 and measure their outcomes among the patients from these years.

## 2. Methodology

### 2.1. Study Design

Here, we present a retrospective cohort study that measures the outcomes among TBI patients in two periods: before the BTF guidelines were implemented (2012) and after (2022). In order to achieve that, we have reviewed all of the patients admitted to our surgical ICU in KKUH, Riyadh, Saudi Arabia, with a diagnosis of TBI, over a period between January 2011 and December 2012, and compared it to TBI patients admitted from January 2021 to December 2022, in order to compare and contrast the effects of the implementation of BTF guidelines and their effect on patient outcomes in a tertiary care center. Furthermore, the patients were identified through prospectively maintained ICU databases in both cohort years. Their medical records were reviewed based on demographics (age, gender, and nationality) and injury information (initial systolic blood pressure (SBP), heart rate (HR), respiratory rate (RR), oxygen saturation (SpO2%), and Glasgow Coma Scale Score (GCS)). Moreover, the outcomes of the patients included their ICU length of stay (LOS), hospital LOS, and mortality. In addition to that, the injury severity score (ISS) was calculated for all patients. In both cohorts, we included patients ranging from 18 to 60 years of age, who presented with a GCS of 8 or lower, irrespective of other characteristics, and who were admitted to the surgical ICU either directly from the ED or after undergoing a surgical intervention. In both cohorts, the patients who had a GCS higher than 8, or who were pronounced dead on arrival (DOA) were excluded, along with those who died within the first 6 h of arrival to the emergency department. We excluded these patients to increase the reliability of our results. Moreover, approval was granted for the study from our Institutional Review Board (IRB) before commencement, with IRB project number E-23-7985, and all patients’ personal data were omitted and anonymized to ensure the patients’ privacy and safety.

### 2.2. Adherence to Guidelines

The patients’ charts were reviewed and the adherence to management guidelines was examined according to Lee and his colleges’ [16] definition of the compliance to BTF guidelines. The definition was followed for both cohorts in our approach, and was slightly modified to ensure the detection of the true percentage of noncompliance. The modifications included the search for hypoxia and hypotension in the whole hospital length of stay for the patients, compared to the suggested 24 or 48 h from the patient’s admission. These modifications were revised to obtain justification and approval by trauma surgeons and intensivists.

Blood pressure and oxygenation, avoidance of hypotension and hypoxia: The BTF guidelines recommend monitoring blood pressure and oxygen saturation to avoid a drop in systolic blood pressure below 90 mmHg and a drop in oxygen saturation below 90%. Patients with a drop in either of these parameters during their whole stay in the ICU, without a reversal of the drop within two hours, were recorded as noncompliance to guidelines.

Hyperosmolar therapy: The guidelines recommend using mannitol at a dose of 0.25–1 g/Kg or using hypertonic saline (3%) till the sodium is raised and maintained at 145 m/Eq, to control increased intracranial pressure (ICP) in TBI patients. The use of either mannitol or hypertonic saline was documented.

Deep Venous Thromboembolism (DVT) prophylaxis: the guidelines recommend the initiation of DVT prophylaxis (graduated compression stockings or intermittent compression devices and pharmacological prophylaxis when possible) in patients with severe TBI.

ICP monitoring: The guidelines recommend monitoring ICP in patients with a severe TBI and an abnormal brain CT scan (defined as a scan of the head revealing hematomas, contusions, swelling, herniation, or compressed basal cisterns) and in patients with severe TBI and a normal brain CT scan if they are above 40 years old, have motor posturing, or have a systolic blood pressure below 90 mmHg. The indications were the main determinators whether the monitoring was indicated by the guidelines. Whenever that was the case it was documented as “compliance with guidelines”. When the abovementioned points were met in a patient who did not receive ICP monitoring they were labeled as “non-compliance”.

Antiseizure Prophylaxis: the guidelines recommend the use of antiseizure prophylaxis to prevent early posttraumatic seizure within the first 7 days after the trauma.

All the above-mentioned parameters were observed for compliance with BTF guidelines within the first 24 h of the patient’s presentation, except for avoidance of hypotension and hypoxia and ICP monitoring (as detailed prior); those parameters were measured for the whole admission period.

### 2.3. Statistical Analysis

A statistical analysis was performed using SPSS, version 29 (SPSS, Chicago, IL, USA). Descriptive statistics in the form of mean, media, and standard deviation were included. Continuous variables were compared using the Student’s *t* test, while categorical variables were compared using the Chi-square test. The level of significance was considered at a *p* value of less than 0.05.

## 3. Results

### 3.1. Demographics

The number of patients admitted to our ICU with a diagnosis of a severe TBI in the 2012 cohort was 33 patients, compared to 49 patients in the 2022 cohort. The mean age of the patient population was 29.5 years and 30.3 years, respectively. The majority of the injured patients in both cohorts were Saudi, and almost all of them were males.

Table 1 summarizes the demographics of the TBI patients, comparing both cohorts.

### 3.2. TBI Related Parameters

The mean ISS was 27.4 in the 2012 cohort; similarly, the 2022 cohort had a mean ISS of 27.1. A pattern of improvement was noticed in the mean GCS—4.82 in 2012 compared to 5.6 in 2022. The same was observed regarding the mortality rate as it decreased from 42.4% in 2012 to 16.3% in 2022. Upon presentation to the emergency department, both cohorts had very similar vitals, with a noticeable increase in SpO2% in the 2022 cohort. For the 2012 cohort the mean HR was 106.2 bpm, the mean SBP was 131.7 mmHg, and there was no difference between the amount of dead or alive patients after the initial HR or SBP measurement. A lower mean GCS upon presentation was significantly associated with death (3.8 vs. 5.7 in the alive cohort); the median GCS in patients who lived was 6, while it was 3 in patients who died.

Compared to the 2012 cohort, the 2022 cohort had a mean HR of 107.4 bpm, mean SBP of 118.1 mmHg, and higher mean SpO2% of 92.1%, but there was no association between SpO2% and mortality. The GCS of the 2022 cohort was not significantly associated with mortality.

Table 2 summarizes the TBI parameters and initial vitals for the 2012 cohort and the 2022 cohort, respectively.

### 3.3. BTF Guidelines Implementation

The rate of adherence to the BTF guidelines was as follows in the 2012 cohort: hypotension and hypoxia were avoided in 42.4% and 60.6% of patients, respectively. ICP monitoring was performed in 36.4% of patients. Hyperosmolar therapy was initiated in 51.5% of patients; furthermore, it should be noted that all patients treated in the KKUH had been treated by hypertonic saline, as mannitol was rarely used in our center. DVT prophylaxis was initiated in 81.9% and antiseizure prophylaxis was initiated in 78.1% of the patients.

Compared to the 2012 cohort, in 2022 hypotension and hypoxia were avoided in 81.1% and 100% of patients, respectively. ICP monitoring was performed in 18.7% of patients and hyperosmolar therapy was initiated in 79.1% of patients. DVT prophylaxis was performed in 77.0% of patients and seizure prophylaxis was initiated in 83.3% of patients.

Table 3 summarizes the guideline implementation in our TBI patients in 2012 and 2022, respectively.

### 3.4. Outcomes

In the 2012 cohort, the mean ICU LOS was 15.9 days, and the mean hospital LOS was 27.33 days, with a mortality rate of 45.4%. In contrast, the 2022 cohort’s mean ICU LOS was 11.7 days, and the mean hospital LOS was 49.2 days, with a mortality rate of 14.2%. Furthermore, the patients were divided into two cohorts based on the outcome (dead or alive) in both of the cohorts (2012 and 2022).

Table 4 compares the outcomes of both of our cohorts and two other local trauma centers. Table 5 shows multiple other studies from the Middle East showing the mortality rate of TBI patients.

## 4. Discussion

When comparing the two periods (2012 and 2022), the current study found that in 2022 there was a 33% increase in the incidence of traumatic brain injuries (TBIs) in our hospital, compared to 2012. This significant increase could be attributed to multiple factors, such as enhanced protocols and more trauma patients to being taken to level one trauma centers that are able to provide the clinical service needed, instead of being admitted to the nearest hospital [18,19]. Despite the increased number of severe TBIs arriving at our hospital, the injury severity score (ISS) mean remained similar in the two periods, showing a similar level of injury severity despite the stronger anti-traffic violation and road safety measures, such as automated violations for drivers using their mobile phones and not wearing seat belts while driving [20].

Adherence to the evidence-based guidelines remains crucial during practice, as shown by the significant decrease in mortality rates, from 42.2% in 2012 to 16.3% in 2022. Avoidance of hypoxia and hypotension are two major predicators of survival in severe TBI patients. Better adherence to the Brain Trauma Foundation (BTF) guidelines has led to the reduction in these two events as they were avoided in most of the patients with severe TBI in the 2022 cohort (100% avoidance of hypoxia and 81.1% avoidance of hypotension). Hyperosmolar therapy was also utilized more, and we noticed a 20% increase in usage by 2022. These are some of the factors that collectively contributed to the improved outcomes. On the contrary, there was a significant gap in adhering to specific elements of the BTF guidelines, notably the intracranial pressure (ICP) monitoring, which is reported to be 77.4% in the literature, whereas in our institution only 36.4% of patients had their ICP monitored. We believe this is mainly due to the neurosurgeons’ resistance to inserting ICP monitors due to conflicting evidence of their benefit in trauma patients [21,22]. Interestingly, ICP monitoring did not significantly impact the patient outcomes between the two cohorts.

When comparing our 2012 outcomes to those of other local and international trauma centers, it is evident that the lack of formal protocols and standardized clinical practices contributed to worse outcomes. Several key factors have since driven significant improvements in patient care, aligning our institution with centers that have successfully implemented protocolized approaches. An example in our institution is the development of a separate critical care medicine department ran by fellowship-trained ICU physicians (versus anesthesiologists with no specific critical-care medicine training back in 2012) and the initiation of a Trauma and Acute Care Surgery unit. This unit admits all multi-injured trauma patients and is covered only by General Surgeons who have completed fellowship training in trauma and acute care surgery.

Given the global findings emphasizing the importance of protocolized TBI management, studies such as those by [15,23] Fakhry et al. (2005) and Hesdorffer et al. (2007) have demonstrated that the adherence to evidence-based protocols significantly reduces mortality and improves patient outcomes. However, our study was limited by its retrospective design, which prevented the comprehensive exploration of barriers to protocol compliance and limited our ability to analyze these factors in depth.

Several limitations of our study should be acknowledged. First, the retrospective design of the study, which often has inherent biases, such as selection bias and the potential for incomplete or inaccurate data, which may affect the reliability of our findings. Second, some of the severe TBI patients who did not reach the ICU were excluded from the study, potentially affecting our findings. Finally, while some of the reasons for not adhering to the protocolized approach were explored, not all were identified. A more comprehensive analysis would have provided valuable recommendations for future improvement.

The positive impact of BTF guideline implementation observed in our study highlights the need for further research to sustain and build upon these improvements. A prospective, multicenter study could validate our findings, reduce biases, and provide a more comprehensive assessment of guideline adherence. Additionally, future research should focus on identifying and addressing the barriers to protocol compliance, particularly ICP monitoring and other critical interventions. Studying Saudi Arabia’s implementation strategies and comparing them to international best practices may help refine local guidelines and enhance patient care. Ultimately, these efforts will not only improve patient outcomes in Saudi Arabia, but also contribute to global advancements in severe TBI management, particularly in optimizing adherence to evidence-based protocols.

## 5. Conclusions

TBI is a severe devastating condition that results from trauma. Managing such patients should be meticulous, with very detailed control of the patients’ parameters in ICU settings. Therefore, BTF guidelines were created, and then updated when there was enough evidence proving the enhancement in the patients’ outcomes. Multiple different studies show proof of a huge improvement in the patients’ outcomes when these guidelines were applied and followed. In this study, we discovered the reasons for implementing these guidelines and the negative effect of the lack of their implementation in our tertiary care center. We noticed a 25% reduction in mortality when these guidelines were applied, with an improvement to all the other parameters. Moreover, the reasons for the lack of the implementation were discussed and could be attributed to the poor-quality control in our center, which became better with time, to specialists managing the patients instead of consultants, and to unclear hospital policy.

## Figures and Tables

**Table 1:** Summary of the demographics of our population, TBI parameters and initial vitals for the 2012 and 2022 cohorts respectively.

		2012 Cohort				2022 Cohort			
		Severe TBI	Survived	Mortality	*p* Value	Severe TBI	Survived	Mortality	*p* Value
Number of Patients		33	18	15	NA	49	42	7	NA
Age, mean ± SD, years		29.5 (17.7)	26.7 (14.5)	32.8 (20.8)	0.35	30.3 (9.1)	30.2 (8.57)	30.7 (12.5)	0.929
Saudi Nationality, (%)		63.6%				40.8%			
Male Gender, n (%)		32 (97)	17 (94.4)	15 (100)	NA	46 (93.8%)	92.60%	7 (100%)	NA
Vital signs at arrival	HR mean ± SD	106.2 (31.2)	108.7 (28.2)	103.6 (34.7)	0.66	107.4 (39.4)	104.8 (41.9)	120.5 (19.1)	0.327
	SBP mean ± SD	131.7 (29.3)	126.8 (18.2)	137.1 (37.7)	0.35	118.1 (39.2)	112.4 (35.2)	146.8 (48.1)	0.022
	RR mean ± SD	25.3 (8.3)	24.7 (6.5)	26 (10)	0.68	27.0 (9.4)	27.0 (9.5)	27.1 (9.8)	0.462
	SPO2 mean ± SD	89.7 (10.7)	94.1 (7.7)	84.5 (11.7)	0.02	92.1 (8.5)	91.8 (8.7)	93.7 (8.1)	0.545
GCS	Mean ± SD	4.82 (1.9)	5.7 (2)	3.8 (1.4)	0.003	5.6 (2.0)	5.6 (2.0)	5.6 (1.9)	0.95
ISS mean ± SD		28.8(13.2)	24.8(11.9)	33.7(13.4)	0.054	27.1 (19.2)	25.1 (15.2)	36.8 (32.3)	0.505
Mortality, n (%)		15 (42.4)				7 (14.2)			

**Table 2:** The guidelines implementation in our TBI patients in 2012 and 2022, respectively.

	2012 Cohort				2022 Cohort			
	Severe TBI	Survived	Mortality	*p* Value	Severe TBI	Survived	Mortality	*p* Value
Hypotension, n (%)	19 (57.6)	5 (27.8)	14 (93.3)	<0.001	9 (18.3)	7 (17.0)	2 (25)	<0.001
Hypoxia, n (%)	13 (39.4)	1 (5.6)	12 (80)	<0.001	0 (0)	0 (0)	0 (0)	<0.001
Hyperosmolar Therapy, n (%)	17 (51.5)	8 (44.4)	9 (60)	0.37	38 (77.5)	31 (75.61)	7 (87.5)	<0.001
DVT Prophylaxis, n (%)	29 (87.9)	18 (100)	11 (73.3)	0.02	24 (81.9)	20 (48.7)	4 (50)	<0.001
ICP Monitoring, n (%)	12 (36.4)	6 (33.3)	6 (40)	0.69	3 (6.1)	0 (0)	3 (37.5)	<0.001
Antiseizure Prophylaxis, n (%)	25 (75.8)	14 (77.8)	11 (73.3)	0.77	33 (67.3)	28 (68.2)	5 (62.5)	<0.001

**Table 3:** The guideline implementation rate in our TBI patients in 2012 and 2022 cohorts, re-spectively.

	Implementation Rate 2012	Implementation Rate 2022
Avoidance of hypotension	42.4%	81.1%
Avoidance of hypoxia	60.6%	100%
Hyperosmolar therapy	51.5%	77.5%
DVT prophylaxis	87.9%	81.9%
ICP monitoring	36.4%	6.1%
Antiseizure prophylaxis	75.8%	67.3%

**Table 4:** A comparison between the outcomes of both of our cohorts and two other local trauma centers.

	2012 Cohort	2022 Cohort	Haddad et al. [12]	Arabi et al. [11]
Number of Patients	33	49	477	362
Age, mean ± SD, years	29.4 (17.7)	30.3 (9.1)	29.3 (14)	29.5 (14)
Male Gender	97%	93.8%	95.8%	94.9%
Mean GCS ± SD	4.8 (1.9)	5.6 (2.0)	5 (1.9)	4.9 (1.8)
Mean ISS ± SD	29 (13.2)	27.1 (6.6)	30 (11.2)	31.6 (11.5)
Mean ICU LOS ± SD, days	15.9 (14.4)	11.7 (8.2)	11.6 (8.3)	11.9 (7.9)
Mean Hospital LOS ± SD, days	27.3 (27.8)	49.2 (102.5)	78.4 (33.2)	71.4 (79.1)
Mortality, n (%)	15 (42.4%)	7 (14.2%)	15.3%	18.8%

**Table 5:** Shows multiple other studies from the Middle East showing the mortality rate of TBI patients.

Studies [17]	Population		Mortality
El-Matbouly et al. (2013)	1665 TBI patients, all severe.		11%
Al-Kashmiri et al. (2015)	174 TBI patients, intensive care unit-admitted TBI, conservative treatment. Exclusion: surgical treatment.	Median GCS in two cohorts (3 and 5)	20%
Haddad et al. (2011)	477 severe TBI patients, adult (≥18 year) patients with TBI admitted to intensive care units.	All were severe cases.	In-hospital mortality, 16% vs. ICU TBI mortality, 12%
Al-Dorzi et al. (2015)	101 isolated TBI patients, isolated TBIs. Exclusions: age < 14 years, other significant organ injuries, bleeding from other sites, penetrating head injuries, chronic anemia, death or brain death < 24 h post-TBI.	Severe TBI (GCS ≤ 8) in 80%	In hospital mortality 14.9%
Younis et al. (2011)	312 TBI patients, TBI as diagnosis; all severe. Exclusion: insufficient data.	GCS: mild (13–15) 54%	7%
Shehata et al. (2011)	101 patients with isolated head trauma. Exclusion: history of hepatic or hemorrhagic diseases, lethal brain injury or brain herniation, patients on anticoagulation medication.	Mean GCS, 6 ± 2 (range 3–14); APACHE II score: 15 ± 5 (range 3–30)	45.5%

## Data Availability

The original contributions presented in this study are included in the article. Further inquiries can be directed to the corresponding author.

## References

[1] Shackford S., Mackersie R., Holbrook T., Davis J., Hollingsworth-Fridlund P., Hoyt D., Wolf P.L. (1993). The epidemiology of traumatic death. A population-based analysis. Arch. Surg..

[2] Peden M., McGee K., Krug E. (2002). Injury: A Leading Cause of the Global Burden of Disease, 2000.

[3] Boto G.R., Gomez P.A., De La Cruz J., Lobato R.D. (2006). Severe head injury and the risk of early death. J. Neurol. Neurosurg. Psychiatry.

[4] Rutland-Brown W., Langlois J.A., Thomas K.E., Xi Y.L. (2006). Incidence of traumatic brain injury in the United States, 2003. J. Head Trauma Rehabil..

[5] Thurman D.J., Alverson C., Dunn K.A., Guerrero J., Sniezek J.E. (1999). Traumatic brain injury in the United States: A public health perspective. J. Head Trauma. Rehabil..

[6] Al-Shareef A.S., Thaqafi M.A., Alzahrani M., Samman A.M., AlShareef A., Alzahrani A., Alzahrani A., Rio A., Hariri B., Ramadan M. (2022). Traumatic Brain Injury Cases’ Mortality Predictors, Association, and Outcomes in the Emergency Department at a Tertiary Healthcare Center in Saudi Arabia. Asian J. Neurosurg..

[7] (2012). Health Statistical Yearbook.

[8] World Health Organization: WHO Reducing Road Crash Deaths in the Kingdom of Saudi Arabia. https://www.who.int/news/item/20-06-2023-reducing-road-crash-deaths-in-the-kingdom-of-saudi-arabia.

[9] Carney N., Totten A.M., O’Reilly C., Ullman J.S., Hawryluk G.W., Bell M.J., Bratton S.L., Chesnut R., Harris O.A., Kissoon N. (2007). Guidelines for the Management of Severe Traumatic Brain Injury. J. Neurotrauma.

[10] Carney N., Totten A.M., O’Reilly C., Ullman J.S., Hawryluk G., Bell M.J., Bratton S.L., Chesnut R.M., Harris O.A., Kissoon N. (2016). Guidelines for the Management of Severe Traumatic Brain Injury, Fourth Edition. Neurosurgery.

[11] Arabi Y.M., Haddad S., Tamim H.M., Al-Dawood A., Al-Qahtani S., Ferayan A., Al-Abdulmughni I., Al-Oweis J., Rugaan A. (2010). Mortality reduction after implementing a clinical practice guidelines-based management protocol for severe traumatic brain injury. J. Crit. Care.

[12] Haddad S., Aldawood A., Alferayan A., Russell N., Tamim H., Arabi Y. (2011). Relationship between intracranial pressure monitoring and outcomes in severe traumatic brain injury patients. Anaesth. Intensive Care.

[13] Gerber L.M., Chiu Y.L., Carney N., Härtl R., Ghajar J. (2013). Marked reduction in mortality in patients with severe traumatic brain injury. J. Neurosurg..

[14] Song S.Y., Lee S.K., Eom K. (2016). Analysis of mortality and epidemiology in 2617 cases of traumatic Brain Injury: Korean Neuro-Trauma Data Bank System 2010–2014. J. Korean Neurosurg. Soc..

[15] Hesdorffer D.C., Ghajar J. (2007). Marked improvement in adherence to traumatic brain injury guidelines in United States trauma centers. J. Trauma.

[16] Lee J.C., Rittenhouse K., Bupp K., Gross B., Rogers A., Rogers F.B., Horst M., Estrella L., Thurmond J. (2015). An analysis of Brain Trauma Foundation traumatic brain injury guideline compliance and patient outcome. Injury.

[17] El-Menyar A., Consunji R., Al-Thani H., Laher I. (2021). Traumatic Brain Injury in the Arab Middle East. Handbook of Healthcare in the Arab World.

[18] Palmer S., Bader M.K., Qureshi A., Palmer J., Shaver T., Borzatta M., Stalcup C. (2001). The impact on outcomes in a community hospital setting of using the AANS traumatic brain injury guidelines. Americans Associations for Neurologic Surgeons. J. Trauma.

[19] Patel H.C., Menon D.K., Tebbs S., Hawker R., Hutchinson P.J., Kirkpatrick P.J. (2002). Specialist neurocritical care and outcome from head injury. Intensive Care Med..

[20] DeNicola E., Aburizaize O.S., Siddique A., Khwaja H., Carpenter D.O. (2016). Road Traffic Injury as a Major Public Health Issue in the Kingdom of Saudi Arabia: A Review. Front. Public Health.

[21] Tang A., Pandit V., Fennell V., Jones T., Joseph B., O’Keeffe T., Friese R.S., Rhee P. (2014). Intracranial pressure monitor in patients with traumatic brain injury. J. Surg. Res..

[22] Florez-Perdomo W.A., Mishra R., Moscote-Salar L.R., Cincu R., Maurya V.P., Agrawal A. (2024). Intracranial Pressure Monitoring in Patients with Traumatic Brain Injury: An Umbrella Review of Systematic Review and Meta-Analysis. J. Neurointensive Care.

[23] Fakhry S.M., Trask A.L., Waller M.A., Watts D.D. (2004). Management of brain-injured patients by an evidence-based medicine protocol improves outcomes and decreases hospital charges. J. Trauma.

